# Virulence factors and outcomes in bacteremia caused by extended-spectrum β-lactamase-producing uropathogenic *Escherichia coli* ST131-H30Rx in a Swedish county

**DOI:** 10.1186/s12879-025-11519-9

**Published:** 2025-09-08

**Authors:** Vidar Möller, Jenny Welander, Maud Nilsson, Mai Thi-Huyen Nguyen, Martin Holmbom, Håkan Hanberger, Lennart E. Nilsson, Åse Östholm, Björn Berglund

**Affiliations:** 1 Department of Infectious Diseases in Östergötland, Department of Biomedical and Clinical Sciences, Linköping University, Linköping, Sweden; 2 Department of Clinical Microbiology, Department of Biomedical and Clinical Sciences, Linköping University, Linköping, Sweden; 3 Department of Biomedical and Clinical Sciences, Linköping University, Linköping, Sweden; 4 Department of Protein Science, KTH Royal Institute of Technology, SciLifeLab, Stockholm, Sweden; 5 Department of Urology in Östergötland, Department of Biomedical and Clinical Sciences, Linköping University, Linköping, Sweden; 6 Department of Cell and Molecular Biology, Uppsala University, Uppsala, Sweden

**Keywords:** Antimicrobial resistance, *Escherichia coli*, ST131, Sepsis, Extended-spectrum β-lactamase

## Abstract

**Background:**

*Escherichia coli* ST131 and clade H30Rx are the most prevalent extended-spectrum β-lactamase-producing *E. coli* (ESBL-EC) causing bacteremia and urinary tract infections globally and in Sweden. Previous studies have linked ST131-H30Rx with septic shock and mortality, as well as prolonged carriage. In our previous study, ST131 constituted 54% of all ESBL-EC bacteremia originating from the urinary tract.

**Method:**

Utilizing whole-genome sequencing, we retrospectively compared virulence factors (VFs) and patient outcomes based on medical records among 77 isolates of ESBL-EC from 76 patients with pyelonephritis between 2009 and 2018 in a Swedish county.

**Results:**

The VFs Ibes and uropathogenic specific protein were associated with ST131 of all clades (*p* < 0.0001). Serine protease (*p* < 0.0001) and *cnf1* (*p* = 0.0003) were more common among ST131-H30Rx compared to non-ST131 isolates whereas enterobactin and *iss* were more common among ST131-H30Rx compared to both other ST131 isolates (*p* < 0.0001 and *p* = 0.0007, respectively) and non-ST131 isolates (*p* < 0.0001). Sepsis within 36 h was less common among patients infected with ST131-H30Rx (*p =* 0.038).

**Conclusions:**

ST131-H30Rx isolates carried VFs which were associated with recurrence but not uniformly to sepsis. In this explorative study, our results indicate that the ST131-H30Rx clade are not more prone to cause severe infection than other sequence types, but prone to cause recurrence, in addition to ESBL production which limits treatment options. Further studies are warranted to explore the mechanisms driving the success of ST131-H30Rx isolates in causing recurrent infections and colonization, and to form preventive measures.

**Supplementary Information:**

The online version contains supplementary material available at 10.1186/s12879-025-11519-9.

## Introduction

The global burden of antimicrobial resistance (AMR) has been estimated to cause between 1.1 and 1.3 million deaths per year directly attributable to and between 4.2 and 5.2 million deaths associated with antibiotic-resistant bacteria [1]. Both globally and in Sweden, bacteremia and less severe infections with antibiotic-resistant bacteria are most commonly caused by the gram-negative bacterium *Escherichia coli* [2–4]. 3rd -generation cephalosporins are the first-line antibiotics used for empirical treatment of infections caused by *E. coli*, however, pathogenic strains are frequently resistant to broad spectrum antibiotics. The prevalence of extended-spectrum ß-lactamase-producing *E. coli* (ESBL-EC) which are resistant to 3rd -generation cephalosporins, increased in Sweden between 2001 and 2015, but has since then remained stable and currently constitutes 7.6% of all *E. coli* in blood cultures [4]. 

The global increase of ESBL-EC during the last decades has partly been caused by the clonal expansion of *E. coli* sequence type (ST) 131 [5]. A subgroup of ST131 isolates, clade H30Rx, has been associated with mortality and sepsis [6, 7]. In addition to resistance genes, ST131 isolates also carries virulence factors (VFs). These are bacterial traits enhancing the capacity to cause disease and invade different environments in the host, such as the gut or the bladder. VFs are encoded by genes which can be carried together with antibiotic resistance genes on mobile genetic elements such as plasmids [8]. Infections with bacteria carrying VFs may influence patient outcomes in terms of both sepsis [9, 10] and mortality [6]. In our previous study on adult patients in Östergötland County, Sweden, with urine and blood cultures positive for ESBL-EC between 2009 and 2018, we found that ST131 isolates constituted 54% of all ESBL-EC [11]. The rate of patients with sepsis at presentation according to Sepsis 3 criteria was 51%, while the mortality was low at 3%. Out of 37 episodes of sepsis among the patients, 19 were caused by ST131 isolates. Utilizing whole-genome sequencing, we thus performed this continuation study to characterize this epidemic high-risk ESBL-EC clade of isolates in terms of carriage of VFs and association with sepsis.

## Materials and methods

### Study population and strain collection

The study population and data collection has been described in our previous study [11]. In short, all adults with a blood and urine sample positive for ESBL-EC within 7 days from each other between January 1 st 2009 and December 31 st 2018 in Östergötland County, Sweden, were included in the study. ESBL-EC screening was performed by the Microbiology Department as a part of the clinical routine at Linköping University Hospital by using disc diffusion for cefotaxime and ceftazidime as indicators. Isolates positive in ESBL-screening were subsequently frozen and stored in −80 °C. A total of 77 episodes of bloodstream infections caused by ESBL-EC in 76 patients were identified by using the database of the Microbiology Department. One isolate from each episode was thawed from storage in −80 °C and included in the study. Clinical data from all patients included in the study was obtained from medical records. Antibiotic susceptibility testing was performed with disc diffusion according to EUCAST guidelines.

### Definitions

Sepsis was defined as an increase in Sequential Organ Failure Assessment (SOFA) Score of 2 points or more. Mortality was defined as all-cause 30-day mortality. When comparing antibiotic susceptibility, “Area of technical uncertainty” has been classed as resistant, while “Sensitive, Increased exposure” has been classed as susceptible.

### Whole-genome sequencing

All 77 ESBL-EC isolates from 76 patients were whole-genome sequenced as part of our previous study (Holmbom et al.). In short, extraction of the DNA was performed by using the EZ1 DNA Tissue Kit (Qiagen, Hilden, Germany) according to the manufacturer’s instruction, and 20 ng of extracted DNA was subsequently used to construct a sequencing library. The library preparation was performed with the QIAseq FX DNA Library Kit (Qiagen, Hilden, Germany) according to the manufacturer’s instructions. A Qubit 2.0 fluorometer (Thermo Fisher Scientific, Waltham, MA) and a QIAxcel instrument (Qiagen, Hilden, Germany) were utilized for DNA quantification and quality assurance. Finally, whole-genome sequencing with 2 × 300 bp paired-end reads was carried out on a MiSeq instrument (Illumina, San Diego, CA).

### Bioinformatic analysis

Virulence genes were identified by using an in-house database based on VirulenceFinder (https://cge.food.dtu.dk/services/VirulenceFinder/) and Virulence Factor Database (http://www.mgc.ac.cn/VFs/). A match was considered true if nucleotide identity and coverage were > 90%. A total of 270 virulence genes were identified among all isolates, and grouped in operons. An operon was considered functional in isolates carrying at least 75% of the genes of that operon. ST was determined by multi-locus sequence typing (MLST) by using the Achtman 7 gene scheme [12]. O-serotype was determined in silico by using the Center for Genomic Epidemiology online tool SerotypeFinder 2.0 [13], with 85% threshold for identity and 60% threshold for minimum length. PlasmidFinder 2.1 [14] (https://cge.food.dtu.dk/services/PlasmidFinder/) was used to identify Incompatibility (Inc) groups.

### Phylogenetic relationship and identification of ST131-H30 clades

Phylogenetic trees based on single-nucleotide polymorphisms (SNP) were constructed in CLC Genomics Workbench v. 24.0.2 with the Microbial Genomics Module v. 24.1.1 (Qiagen, Hilden, Germany). Sequencing reads were mapped to the *E. coli* NCBI reference genome NC_022648. Variants were called in relation to the reference genome with the following thresholds: frequency ≥ 90%, sequencing depth ≥ 20x and quality (Phred) score ≥ 20 at the variant position and ≥ 15 in the +/- 5 bp neighborhood. Identified variant positions were filtered based on a sequencing depth of ≥ 20x in all samples, a Z-value ≥ 1.96 and a pruning distance of 10 bp. The resulting positions were used to build neighbor-joining phylogenetic trees (one for all samples and one for ST131 only) based on the genetic distance between samples. Based on previous studies [15], isolates differing by ≤ 15 SNPs were regarded as clonally related.

ST131 clades H30 and H30Rx were determined by using a method based on SNPs (available from: https://urn.kb.se/resolve?urn=urn:nbn:se:his:diva-22867.). The method was first proposed by Banerjee et al. [16]. In brief, the following process was employed: first MLST was determined to establish that the isolate belonged to ST131. Secondly, *fimH* allele detection was performed by using the FimTyper tool based on Basic Local Alignment Search Tool with a minimum threshold for identity 95% and a minimum query cover threshold of 60%. The last step was variant calling for analysis of SNPs according to Freebayes, to identify SNP G723A in gene *ybbW* as an H30Rx biomarker.

Virulence gene analysis was also performed on three previously described strains of ST131-H30Rx: JJ1186 [17], EC958 [18], and NA097 [19], with accession numbers ASM49375v1, EC958.v1, and ASM102941v1, respectively.

### Statistical analysis

**Fig. 1:**
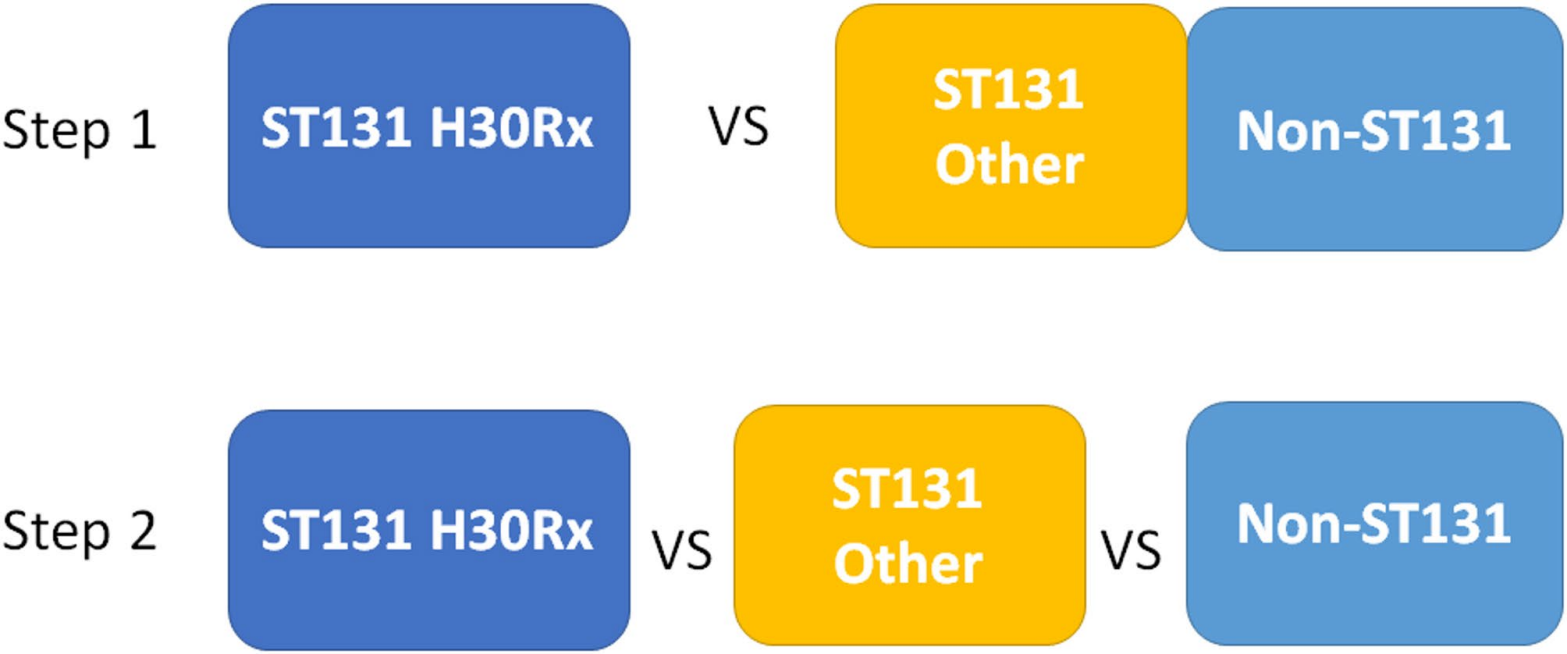
Schematic overview of statistical comparisons of *Escherichia coli* isolated from blood samples from patients with infections originating from the urinary tract. Isolates identified as ST131-H30Rx (dark blue box) were in Step 1 compared to a group consisting of isolates belonging to sequence types (STs) other than ST131 (designated as “Non-ST131” in light blue boxes) and isolates of ST131 which did not belong to the H30Rx clade (designated as “ST131 Other” in yellow boxes). In step 2, the ST131-H30Rx, ST131 Other, and Non-ST131 groups were all compared to each other

Statistical comparisons of virulence genes and patient outcomes were carried out in two steps as outlined in Fig. 1. In the first step, comparisons were made between isolates belonging to ST131-H30Rx and all other isolates. In the second step, comparisons were made between three groups consisting of isolates belonging to ST131-H30Rx, isolates of ST131 not belonging to H30Rx (designated as ST131 Other), and isolates not belonging to ST131 (designated as non-ST131). Patient data were divided similarly according to which isolate each patient was infected with.

Fisher’s exact test was used to identify differences between ST131-H30Rx and ST131 Other and to identify differences in distribution of virulence operons among the three groups a priori and between each separate group *post hoc* for operons which differed from the expected distribution. Operons carried by less than 10% of isolates in all groups were excluded from statistical analysis. To avoid false rejection of the null hypothesis due to multiple comparisons, *p* < 0.001 was considered significant. All statistical analyses were carried out in SPSS v.25.

In a process similar to the one used for virulence operons among the isolates, metadata of patients positive for ST131-H30Rx and ESBL-EC of other STs were compared by using independent samples t-tests, χ^2^-tests, and Mann-Whitney U tests as appropriate. The metadata was further analysed from patients positive for ST131-H30Rx, other ST131, and non-ST131 by using χ^2^-tests or Fisher’s exact test and ANOVA.

Finally, a binary logistic regression model with sepsis at 36 h as dependent variable, was designed including all patient metadata variables with *p* < 0.05. The three variables with lowest *p-*values were included in the final model.

## Results

### Phylogenetic relationship and identification of ST131 H30 clades

A total of 77 isolates of ESBL-EC were whole-genome sequenced and subsequently analyzed in terms of phylogenetic relationship. Average coverage was 73.9. 42 (54.5%) isolates belonged to ST131 with 21 (27.3%) belonging to the clade ST131-H30Rx.

**Fig. 2:**
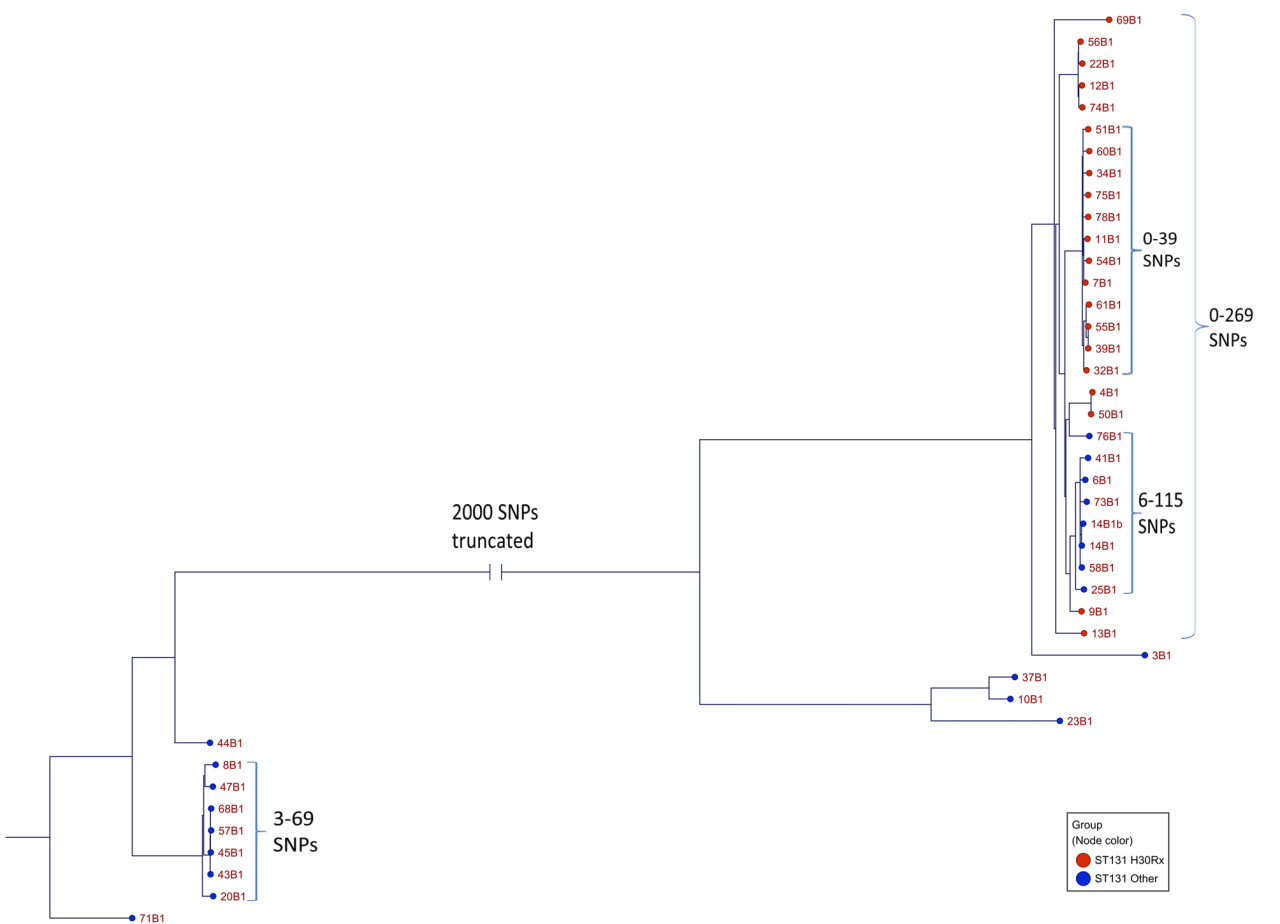
Phylogenetic tree based on single-nucleotide polymorphisms (SNPs) visualizing the genetic relationship among 42 isolates of extended-spectrum β-lactamase-producing ST131 *Escherichia coli* from patients with bloodstream infections originating from the urinary tract. Isolates classified as ST131-H30Rx are denoted with red nodes whereas ST131 isolates not classified as H30Rx are denoted by blue nodes. SNP intervals presented by hook parentheses indicate the minimum and maximum number of SNP differences of isolates within the indicated cluster. To improve the clarity of the figure, a horizontal branch of the phylogenetic tree has been truncated, as indicated by vertical lines. The truncated line represents a difference in SNPs of 2,000.

A phylogenetic tree based on SNPs of the ST131 isolates showed that the majority of isolates grouped together in two clusters (Fig. 2). The larger cluster contained all ST131-H30Rx isolates as well as ST131 isolates not classified as H30Rx. The maximum difference in SNPs among isolates in this cluster was 269 SNPs (between 69B1 and 76B1). Most ST131-H30Rx isolates grouped together, forming a sub cluster with 0–39 SNPs distance between each other. The smallest difference identified among H30Rx isolates was between 55B1 and 39B1 which were identical, and between 4B1 and 50B1 with a distance of 4 SNPs, indicating that these pairs were clonally related. ST131 isolates within the larger cluster which were not classified as H30Rx were all positive for *fim*H30 but did not carry the G723A SNP in *ybbW*. These isolates formed a sub cluster with a maximum difference of 115 SNPs between isolates. The minimum difference was 6 SNPs indicating that isolates 14B1 and 14B1b were clonally related. The smallest difference in terms of SNPs between a non-H30Rx isolate in this sub cluster and a H30Rx isolate was 67 SNPs (between 9B1 and either 58B1 or 14B1). The smaller main cluster consisted of ST131 isolates which were not classified as H30Rx and were distantly related to isolates in the larger main cluster. The smallest difference in terms of SNPs between isolates in the two main clusters was 4,590 SNPs. The maximum difference in SNPs between isolates in the smaller main cluster was 69 SNPs between isolates 8B1 and 20B1. 43B1, 45B1, 57B1, and 68B1 showed the smallest differences between each other, between 3 and 4 SNPs, indicating that these isolates were clonally related.

### Determination of virulence factors

A total of 269 virulence genes were identified among the 77 ESBL-EC isolates, which organized in 41 virulence operons. Virulence operons with significant asymmetrical distributions across groups are shown in Table 1, whereas comparisons for all virulence operons are presented in Supplementary Table 1. The following VFs were significantly more common among ST131-H30Rx isolates compared to all other isolates: invasion of brain endothelial cells (Ibes) (*p* < 0.0001), serine protease (*p* = 0.0005), enterobactin (*p* < 0.0001), uropathogenic specific protein (*p* < 0.0001), and increased serum survival (*p* < 0.0001) which is coded by the single gene *iss*.

**Table 1:** Distribution of virulence factors among extended-spectrum β-lactamase-producing *Escherichia coli* isolates in blood

	ST131-H30Rx (*n* = 21)		ST131 Other (*n* = 21)		Non-ST131 (*n* = 35)	
	*n*	%	*n*	%	*n*	%
Invasion of brain endothelial cells	21	100^a^	21	100^a^	6	17.1
Uropathogenic specific protein	20	95.2^a^	21	100^a^	5	14.3
Cytotoxic necrotizing factor 1	9	42.9^a^	6	28.6	1	2.9
Serine protease	21	100^a^	17	81	18	51.4
Enterobactin	21	100^b^	6	28.6	0	0
Increased serum survival	21	100^b^	12	57.1	19	54.3

^a^Significant difference compared to non-ST131 isolates

^b^Significant difference compared to both ST131 Other and non-ST131 isolates

Distribution of virulence factors (VFs) among 77 extended-spectrum β-lactamase-producing *Escherichia coli* isolated from patients with bloodstream infections originating from the urinary tract belonging to ST131-H30Rx (*n* = 21), ST131 which did not belong the H30Rx clade (ST131 Other) (*n* = 21), and STs other than ST131 (non-ST131) (*n* = 35). Only VFs found to be significantly asymmetrically distributed among any group are included in this table.

When comparing the three groups ST131-H30Rx, ST131 Other, and non-ST131 with each other, the following VFs were found to be significantly asymmetrically distributed: Ibes (*p* < 0.0001), serine protease (*p* = 0.0002), enterobactin (*p* < 0.0001), uropathogenic specific protein (*p* < 0.0001), cytotoxic necrotizing factor (*p* = 0.0005), and *iss* (*p* = 0.0002). Vacuolating autotransporter protein was nearly significant (*p* = 0.0012) – no ST131-H30Rx isolate carried this VF, while it was carried by one ST131 Other and 11 (31.4%) of non-ST131 isolates. Outer membrane hemin receptor (*p* = 0.0011) was also nearly significant, and was present in all ST131 isolates and 71% of non-ST131 isolates. In *post hoc* testing, the VFs Ibes (*p* < 0.0001) and uropathogenic specific protein (*p* < 0.0001) were significantly associated with ST131 of all clades. Serine protease (*p* < 0.0001) and cytotoxic necrotizing factor 1 encoded by the single gene *cnf1* (*p* = 0.0003) were more common among ST131-H30Rx compared to non-ST131. Enterobactin was more common among ST131-H30Rx compared to ST131 Other (*p* < 0.0001) and non-ST131 (*p* < 0.0001). Likewise, *iss* was more common among ST131-H30Rx than ST131 Other (*p* = 0.0007) and non-ST131 (*p* = 0.0001). The following VFs were common across all three groups (> 50% prevalence): outer membrane hemin receptor, curli fibers, *E. coli* common pilus, type I fimbriae, aerobactin, serine protease, iron/manganese transport, and yersiniabactin.

### Comparison between ST131-H30Rx collected in this study and reference strains

When comparing the reference strains of international ST131-H30Rx isolates with the ST131-H30Rx isolates collected in this study, some virulence operons were present in all reference strains and in all of our study isolates: outer membrane hemin receptor, curli fibers, type I fimbriae, aerobactin, serine protease, iron/manganese transport, yersiniabactin, and enterobactin. Some operons were present in all study isolates but absent in the reference strains: increased serum survival, *E. coli* common pilus, and Ibes. The peritrichous flagella and general secretion pathway operons were present among all reference strains but completely absent among the study isolates. TraJ was present among all reference strains but only one study isolate (equaling 4.8% of the group). The entire distribution of virulence operons among the reference strains is presented in supplementary Table 2.

### Distribution of O-serotypes

**Fig. 3:**
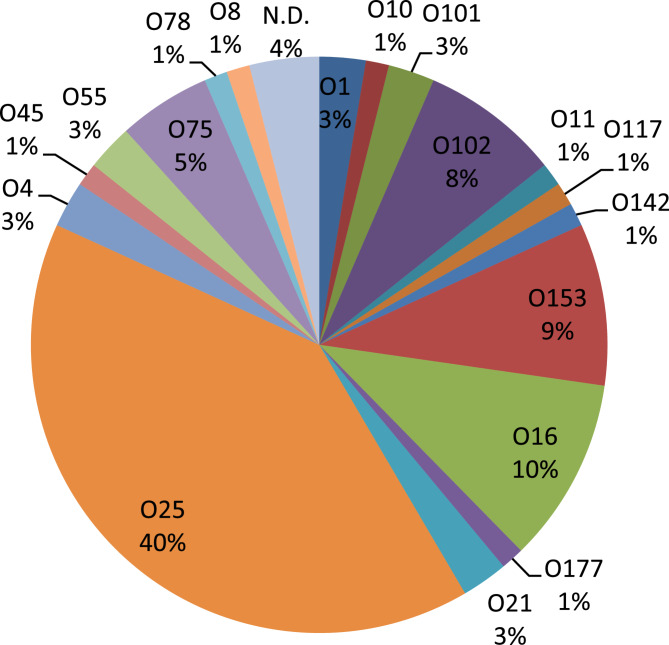
Distribution of O-serotypes among among 77 extended-spectrum β-lactamase-producing *E. coli* isolated from patients with bloodstream infections originating from the urinary tract. Three isolates had undetermined O serotype ((O2/O50, O101/O9, or O17/O44/O77, respectively) and are here shown as non-determinable (N.D.)

The O-serotype of 77 ESBL-EC isolates were analyzed and the most common serotypes were found to be O25 (40%), followed by O16 (10%), O153 (9%), and O102 (8%). The isolates were distributed over a total of 18 different O-serotypes (Fig. 3). In addition, three isolates were indeterminable in terms of O-serotype. These three isolates belonged to O2/O50, O101/O9, or O17/O44/O77, respectively.

### Plasmid analysis

Overall, isolates in this study carried between 0 and 9 incompatibility group replicons. Among ST131 H30-Rx isolates, 81% carried IncFII while corresponding numbers for ST131 Other was 81% and for non-ST131 isolates 74.3%. IncFIA was present in 76.2% of ST131-H30Rx isolates, in 52.4% of ST131 Other, and 40% of non-ST131 isolates. IncFIB was present in 66.7% of ST131-H30Rx, 90.5% of ST131 Other, and 74.3% of non-ST131 isolates. Col156 was present in 47.6% of ST131-H30Rx isolates, in 71.4% of ST131 Other, and 48.6% of non-ST131 isolates. A complete list of Inc-groups is presented in supplementary Table 3.

### Patient background characteristics and outcomes

First, patients with infections caused by ST131-H30Rx were compared to patients infected by other strains (i.e., non-ST130 and ST131 Other). Patients infected by ST131-H30Rx were found to be more likely to be living in nursing homes compared to patients infected by other strains (*p* = 0.044), with 4 (19%) compared to 2 (4%) patients, respectively. Furthermore, sepsis within 36 h occurred among 7 (33%) patients infected by ST131-H30Rx. This was significantly lower (*p* = 0.032) compared to patients infected by other strains, among which 34 (61%) developed sepsis within 36 h. There were no other significant differences between the groups.

Then, patients were divided into three groups and comparisons were made between those infected by ST131-H30Rx, other ST131, and non-ST131. When comparing these three groups, sepsis within 36 h was the only factor for which a significant difference could be observed: specifically, patients infected by ST131-H30Rx were found to be less likely to develop the condition (*p* = 0.038) compared to patients infected by non-ST131 isolates. Demographic data for patients in all three groups is shown in Table 2. Recurring urinary tract infection (UTI) was common among patients in all three groups: 57% in the ST131-H30Rx group, 43% among other ST131 and 37% in the non-ST131 group. Patients in the ST131-H30Rx group received adequate antibiotics 2 h and 21 min quicker in median than non-ST131 patients, but this was not statistically significant (*p* = 0.309). All epidemiological and laboratory data is presented in Supplementary Table 4. Only four patients died among the included cases. Due to the rarity of the event, no statistical analysis was performed to investigate associations with mortality.

**Table 2:** Characteristics and outcomes of patients with bacteremia caused by extended-spectrum β-lactamase-producing *Escherichia coli*

	ST131-H30Rx (*n* = 21)	ST131 Other (*n* = 21)	Non-ST131 (*n* = 35)
Age (SD)	64 (19)	70 (15)	68 (15)
Male (%)	14 (67)	16 (76)	22 (63)
Charlson (SD)	3.1 (2.6)	2.8 (1.7)	2.3 (2.0)
Nursing home (%)	4 (19)	0	2 (6)
Recurrent UTI (%)	12 (57)	9 (43)	13 (37)
Urological cancer (%)	5 (24)	8 (38)	7 (20)
Fluid (%)	14 (67)	19 (91)	27 (77)
Catheterization at presentation(%)	4 (19)	4 (19)	12 (34)
Acute catherterization(%)	12 (57)	10 (48)	18 (51)
Acute nephrostomy/splint (%)	1 (5)	3 (14)	2 (6)
Median time to antibiotics (IQR)	02:35 (01:32 − 04:00)	02:10 (00:50 − 03:05)	02:02 (01:13 − 3:56)
Median time to adequate antibiotics (IQR)	06:34 (03:08–12:06)	07:12 (03:02–17:01)	08:55 (03:18–20:05)
Median days on antibiotics (IQR)	13 (10–18)	14 (12–19)	14 (12–17)
Median hospital days (IQR)	8 (6–11)	7 (5–13)	10 (5–15)
Sepsis at presentation (%)	7 (33)	12 (57)	18 (51)
Sepsis within 36 h (%)	7 (33)	12 (57)	22 (63)*

*SD* Standard deviation, *IQR* Interquartile range, *UTI* Urinary tract infection

*Statistical significance

Clinical and epidemiological variables associated with patients with bacteremia caused by invasion through the urinary tract of extended-spectrum β-lactamase-producing *Escherichia coli* belonging to ST131-H30Rx, ST131 which did not belong the H30Rx clade (ST131 Other), and STs other than ST131 (non-ST131). Patients infected by non-ST131 isolates had a higher frequency of sepsis within 36 h compared to patients infected by ST131-H30Rx (*p* = 0.032).

By utilizing binary logistic regression with sepsis within 36 h as dependent variable, a final model was designed which included the factors ST, sex, and urological cancer (Table 3). Compared to patients infected by ST131-H30Rx, patients infected by non-ST131 had significantly higher frequency of sepsis within 36 h (*p* = 0.038) with an odds ratio of 3.39 (1.07–10.75, 95% confidence interval).

**Table 3:** Multivariate analysis of patients with bacteremia caused by extended-spectrum β-lactamase-producing *Escherichia coli*

Variable	Sig.	OR (95% CI)
Sex	0.283	0.54 (0.18–1.65)
ST131 H30Rx	0.098	Reference
ST131 Other	0.095	3 (0.83–10.91)
Non-ST131	0.038	3.39 (1.07–10.75)
Genitourinary cancer	0.696	1.26 (0.39–4.06)

Multivariate model of clinical and epidemiological variables associated with sepsis at 36 h from admission to hospital among patients with bacteremia caused by invasion through the urinary tract of extended-spectrum β-lactamase-producing *Escherichia coli* belonging to ST131-H30Rx, ST131 which did not belong the H30Rx clade (ST131 Other), and STs other than ST131 (non-ST131) expressed in odds ratio (OR) and 95% confidence intervals (CIs) with ST131-H30Rx as reference.

### Antimicrobial resistance

Among ST131-H30Rx, the rate of ciprofloxacin resistance was 100%, whereas the corresponding numbers among ST131 Other and non-ST131 were 47.6% and 51.4%, respectively. This difference was statistically significant (*p* = 0.000036), and in *post hoc* testing, ciprofloxacin resistance was significantly more common among ST131-H30Rx isolates than among both ST131 Other isolates (*p* = 0.000165) and non-ST131 isolates (*p* = 0.000054). For ceftibuten, resistance rates were 81% among ST131-H30Rx, 23.8% among ST131 Other, and 45.7% among non-ST131 isolates. This difference was statistically significant (*p* = 0.000506), and in *post hoc* testing, ceftibuten resistance was significantly more common among ST131-H30Rx compared to ST131 Other isolates (*p* = 0.000506), but not compared to non-ST131 isolates (*p* = 0.012241). Piperacillin-tazobactam resistance was also more common among ST131-H30Rx isolates (33.4%) than among ST131 Other (14.3%) and non-ST131 isolates (14.3%), however, this difference was not statistically significant (*p* = 0.198). In our study all except two isolates, one each from the groups ST131 Other and non-ST131, were resistant to cefotaxime. No isolate was resistant to meropenem or amikacin. Complete data on AMR and ESBLs are presented in supplementary Table 5.

## Discussion

In this study, we explored VFs among invasive isolates of ESBL-EC originating from the urinary tract. This is a continuation on our previous study where we found that ST131 isolates accounted for 54% of all ESBL-EC causing bacteremia originating from urinary tract infections, most of which carried the ESBL-genes *bla*_CTX−M−14_ or *bla*_CTX−M−15_ [11]. Further analysis of the ST131 isolates in the current study showed that 50% belonged to the clade H30Rx. This is similar to the distribution in other Swedish studies on ESBL-EC bacteremia and UTIs [10, 20, 21]. International studies have found ST131 to account for a similar proportion of ESBL-EC: a Taiwanese study published in 2018 on 843 patients with *E. coli* bacteremia found that 58% of the ESBL-producing isolates belonged to ST131 [22]. In a French multicenter study published in 2022 on ESBL-EC isolates from 213 patients with febrile urinary tract infections and 154 fecal carriage isolates from healthy children, ST131 accounted for 44% in both populations [23]. In the current study, four VFs were associated with the ST131-H30Rx clade isolates specifically: cytotoxic necrotizing factor 1 (encoded by the gene *cnf1*), serine protease, enterobactin, and increased serum survival (encoded by the gene *iss*). Additionally, two VFs were significantly more common among ST131 compared to non-ST131 isolates: Ibes and uropathogenic specific protein.

### Virulence factors, recurrence, and sepsis

A study in Japan comparing genetically identical versus discordant *E. coli* in blood samples from repeated infections found *cnf1* to be more common in isolates that caused recurring infections [24]. *cnf1* modulates cell cytoskeleton and immune response by deamidation of RhoGTPases, affecting caspase-1 activation [25]. 

Serine protease, which causes tissue damage and facilitates entry to the bloodstream, has been inversely associated with sepsis in a prospective study on 103 patients in Belgium with *E. coli* bacteremia [9]. This is in line with our results, where patients with infections caused by ST131-H30Rx isolates were less likely to have sepsis at 36 h. Both serine protease and uropathogenic specific protein have previously been associated with recurrent infections [24] and were significantly more common among ST131-H30Rx isolates than in non-ST131 isolates in the current study.

In a retrospective study on 278 patients with bacteremia caused by ESBL-EC, Fröding et al. found *iss* to be associated with septic shock [10]. This is contrary to our results, where all of the ST131-H30Rx isolates carried *iss*, but patients infected by ST131-H30Rx isolates had significantly lower rates of sepsis within 36 h from admission. However, Fröding et al. did not find any association between ST131-H30Rx isolates and septic shock or death. In addition, in our study, *iss* was common among other ST131 and non-ST131 isolates as well (57% and 54% respectively).

Another VF associated with sepsis is vacuolating autotransporter protein [9] which induces formation of vacuoles in the bladder. This VF was completely absent in the H30Rx group and present in only in one ST131 isolate overall, but found among 11 (31.4%) of the non-ST131 group. The difference was, however, not significant (*p* = 0.0012).

Ultimately, no VF was found to be significantly associated with non-ST131 isolates. This does not necessarily imply that ST131-H30Rx isolates are more virulent than non-ST131 isolates, but could be a result of the study design in which non-ST131 isolates from many different STs were grouped together and their different genotypes might dilute any specific characteristic.

As the study population is small in this study, a difference in sepsis incidence of 20% at presentation did not reach statistical significance. Considering sepsis at 36 h from admission, the difference was roughly 30%, which did reach statistical significance. Consequently, the study size may be too small to detect relevant differences between groups. However, the three groups of patients were quite similar: recurrent UTIs were common in all groups, as was previous antibiotic treatment, and a history of cultures positive for ESBL-producing bacteria.

### Antimicrobial resistance

In our previous study [11] we showed that all isolates included in this study carried genes encoding ESBLs, and the inclusion criteria was resistance to 3rd -generation cephalosporins in clinical routine testing. The two isolates not resistant to cefotaxime in our study may have produced less enzyme when re-evaluated by us, or it may be a result of technical differences, e.g. different agar plate batches or different interpretation of inhibition zones. Ciprofloxacin resistance was significantly more common among ST131-H30Rx isolates than ST131 Other and non-ST131 isolates, which is expected as it is part of the defining features of the ST131-H30Rx clade. In addition, ceftibuten resistance was significantly more common among ST131-H30Rx isolates than ST131 Other, but resistance levels were too high across all three groups for ceftibuten to be a reliable empirical treatment option.

### Uropathogenic *E. coli* virulence factors common in all groups

Aside from increased serum survival (encoded by *iss*), which was common among all isolates included in this study, other VFs were also common across ST131, ST131-H30Rx, and non-ST131 isolates (i.e., > 50% prevalence in all groups). These common VFs included outer membrane hemin receptor, type I fimbriae, curli fibers, *E. coli* common pilus, yersiniabactin, aerobactin, and iron/manganese transport. Carriage of these VFs are common among uropathogenic *E. coli* [26–28], thus the distribution reflects the selection of patients in this study. Among these VFs, aerobactin has notably been inversely associated with sepsis [9] and yersiniabactin has been associated with recurrent infection [24]. In our previous study on the patients from which the ESBL-EC isolates in the current study originated, we found that recurrent UTI was significantly more common among patients infected with ESBL-EC (44%) compared to patients in the control group who were infected by non-ESBL-EC (12%) [11]. Notably, pyelonephritis-associated pilus (containing *papG*-variants) has been associated with bacteremia and febrile UTI in previous studies [23, 29]. In the current study, *papG* was present in 76% of ST131-H30Rx, in 38% among ST131 Other, and in 46% of non-ST131 isolates. However, the differences were not statistically significant. As all isolates in this study were collected from blood samples, a high prevalence of *papG* could be expected.

### Disease and carriage attributed to ST131 and ST131-H30Rx

Previous studies have shown diverging results regarding the clinical outcomes associated with infections caused by ST131 and ST131-H30Rx isolates. In a retrospective study on 843 patients with *E. coli* bacteremia in Taiwan, mortality was higher among patients infected by ST131 isolates [22]. Two Swedish studies, one regarding bacteremia in 278 patients [10] and one regarding urinary tract infection in 235 patients [21] found an association with recurrent infection. In an American study on 1,133 patients with extraintestinal *E. coli* infections, ST131 was associated with a higher frequency of sepsis [7]. However, a Spanish prospective study on 225 patients with sepsis found no differences in septic shock or mortality related to ST [30]. In the current study, recurrent UTI was more common among patients infected by ST131-H30Rx than non-ST131 isolates (57% compared to 37%), however, this difference was not statistically significant. As gut colonization can act as a reservoir for bacteria causing recurrent infections, studies have also investigated whether ST131 is associated with increased duration of gut carriage. A Dutch study on long term care facility residents found ST131 carriage half-life was 13 months compared to 2–3 months for other strains [31]. Similarly, an American study on long-term care facility residents found ST131 carriage to be on average 10 months compared to 3 months for other strains [32]. 

To combat both recurrent UTIs and the spread of antimicrobial resistance, there is an urgent need for interventions, such as vaccines. Among vaccines currently on the market, ExPEC4V containing lipopolysacharide O-antigens from O1A, O2, O6, and O25B would theoretically cover 43% of the isolates in our study. Additionally, a new vaccine, ExPEC10V (currently in phase III trials), would theoretically cover 61% of the isolates in our study.

## Conclusions

Isolates of ST131-H30Rx carried several VFs of which serine protease, *cnf1* and uropathogenic specific protein have been associated with recurrent infections [24]. This is in line with previous studies showing that ST131 isolates and ST131-H30Rx isolates are linked to increased risk for recurrence [20, 21]. The ST131-H30Rx isolates also carried VFs both positively (i.e., increased serum survival) [10] and negatively associated with sepsis (i.e., serine protease) [9]. In the current study, sepsis within 36 h from admission was less common among patients with infections caused by ST131-H30Rx isolates.

These results could indicate that the ST131-H30Rx clade primarily is not more aggressive than other STs. Other studies have indicated that the virulence factors carried by ST131-H30Rx could make it a persistent colonizer in the gut, which is supported by studies on long-term care residents [31, 32]. Recurrent infections were, however, common in our study across all groups, with no statistically significant differences between the groups.

While this was an explorative study, our results may have an impact on vaccines and other future interventions. It is important to investigate which isolates have the potential for severe infections and/or recurrence, so that interventions target important pathogens. There is still a knowledge gap regarding the cause of the increase in ST131-H30Rx isolates. While it is puzzling that sepsis was less common among patients infected with ST131-H30Rx isolates, firm conclusions regarding the clinical data are hard to draw from this limited material. Further studies are needed to elucidate the link between VFs, host factors (e.g., the gut microbiome), and gut carriage of ESBL-EC.

## Supplementary Information

Supplementary Material 1

## Data Availability

The assemblies from whole-genome sequencing are available at NCBI (Accession ID number: SAMN48135659-SAMN48135820).

## Abbreviations

ESBL-EC: Extended-spectrum β-lactamase-producing E. coli
VFs: Virulence factors
AMR: Antimicrobial resistance
ST: Sequence type
MLST: Multi-locus sequence typing
SNP: Single-nucleotide polymorphisms
UTI: Urinary tract infection
